# Cognitive function, depression, and quality of life in patients with ruptured cerebral aneurysms

**Published:** 2018-07-06

**Authors:** Samira Zabyhian, Seyed Javad Mousavi-Bayegi, Humain Baharvahdat, Farhad Faridhosseini, Payam Sasannejad, Maryam Salehi, Maryam Boroumand, Zahra Hatefipour

**Affiliations:** 1 Department of Neurosurgery, Ghaem Hospital, Mashhad University of Medical Sciences, Mashhad, Iran; 2 Department of Psychiatry, Imam Reza Hospital, Mashhad University of Medical Sciences, Mashhad, Iran; 3 Department of Neurology, Ghaem Hospital, Mashhad University of Medical Sciences, Mashhad, Iran; 4 Department of Community Medicine, School of Medicine, Mashhad University of Medical Sciences, Mashhad, Iran

**Keywords:** Subarachnoid Hemorrhage, Cerebral Aneurysm, Cognitive Impairment, Depression, Neuropsychological Test, Quality of Life

## Abstract

**Background:** Neuropsychiatric dysfunction is one of the most common complications after aneurysmal subarachnoid hemorrhage (aSAH). The aim of this study was to evaluate cognitive function, depression, and quality of life (QOL) in patients with aSAH.

**Methods:** In this study, we prospectively enrolled patients with SAH due to rupture of anterior circulation aneurysms who referred to Ghaem hospital, Mashhad, Iran, and who had good function outcome [modified Rankin scale (mRS) > 2]. They underwent microsurgery or endovascular treatment. Cognitive function, depression, and QOL were evaluated 6 months after surgery with standard psychiatric examinations, including Mini-Mental State Examination (MMSE) for cognitive function, Hospital Anxiety and Depression Scale (HADS) for depression, and 36-Item Short Form Health Survey (SF-36) for QOL. Risk factors for cognitive dysfunction were assessed.

**Results:** Fifty-three patients were entered the study. The mean of age was 50.9 ± 13.6 years. QOL and its components were affected in most patients. Fifty-five percent of patients suffered from depression. Cognitive impairment was found in 57% of patients. Older patients experienced more cognitive impairment (P < 0.001).

**Conclusion:** Neuropsychological sequels are common in patients with aSAH, even if they classified as good functional outcome (mRS > 2). These complications could be found with appropriate neuropsychological evaluation of these patients to be managed as soon as possible.

## Introduction

Subarachnoid hemorrhage (SAH) includes only 2-5 percent of all new strokes. It is usually due to rupture of a cerebral aneurysm.^   1 ^ Aneurysmal SAH (aSAH) could lead to a poor outcome and severe disability in many patients. Most studies defined the outcome of patients with aSAH according to modified Rankin scale (mRS) and Glasgow outcome scale (GOS). These scales evaluate the gross functional outcome of these patients, but they cannot assess the cognitive impairment and the quality of life (QOL) of these patients. These details can be attained using specific neuropsychiatric tests. 

During the past two decades, many studies assessed cognitive function and QOL after SAH.^2^^,^^3^ Although the majority of patients with aSAH who have good clinical status at admission experience favorable outcome, more than 50% of them may have cognitive impairment and low QOL.^1^^,^^4^^-^^7^ These patients may also experience personality changes.^        8 ^ In this study, we evaluated cognitive function, depression, and QOL in our patients with aSAH.

## Materials and Methods

This prospective study reviewed the clinical and radiological database of consecutive patients with ruptured aneurysms of anterior circulation who were admitted to Department of Neurosurgery, Ghaem hospital, Mashhad, Iran, between March 2011 and January 2014. The aim of this study was to determine the cognitive function, depression, and QOL in patients with aSAH as the primary endpoint. As the secondary endpoint, we assessed the effect of location, sex, age, and type of treatment on the neuropsychiatric function of these patients.

The inclusion criteria were as: 1) patients with aSAH admitted to the neurosurgical department of Ghaem hospital between March 2011 and January 2014 with ruptured aneurysms, located in anterior circulation confirmed by computed tomography (CT) scan and CT angiography or cerebral digital subtraction angiography, 2) age between 17 and 90 years, and 3) functional recovery allowing the patient to participate in the comprehensive neuropsychological tests (mRS of 0-2).

The exclusion criteria were as: 1) SAH from aneurysm of posterior circulation, 2) SAH due to another cause, 3) previous neurological and psychiatric problems, 4) the patients that could not complete the surveys, 5) previous addiction, 5) pregnancy, and 6) patients who refused to participate.

All patients had been managed by routine medical treatment for SAH. The cerebral aneurysm was occluded by either microsurgery or endovascular approach after multidisciplinary discussion. For each patient, postoperative CT scan and follow-up cerebral digital subtraction angiography were routinely performed. For all patient, neuropsychological tests were performed after 6 months of discharge to evaluate their cognitive function, depression, and QOL.

The neuropsychological test was assessed by a psychologist. She was blinded to the aneurysm location and the type of treatment. QOL was scaled using the 36-Item Short Form Health Survey (SF-36). This questionnaire assessed patients on 8 components: physical functioning (PF), role limitations due to physical health (RP), body pain (BP), general health (GH), mental health (MH), role limitations due to emotional problems (RE), vitality (energy/fatigue) (VT), and social functioning (SF). The first 4 components were also summarized to physical QOL (physical component summary) and the last 4 components to emotional QOL (mental component summary). Physical QOL and emotional QOL were calculated as the mean average of all the physical components and as the mean average of all the mental components, respectively. For each component of SF-36, the standard scores were calculated by dividing the difference between the patient score and the mean reference population score by the standard deviation (SD) of the reference population. The validity and reliability of the SF-36 had been evaluated in the Iranian population previously, and we used the scores of general Iranian population as the reference.^   9^

The level of depression was evaluated by the Hospital Anxiety and Depression Scale (HADS). The HADS has fourteen items, 7 for anxiety and 7 for depression. Each item is scored from 0 to 3 and a person can be scored between 0 and 21. The scores are categorized as follows: normal or borderline, 0-10, and affected, 11-21. We divided the last one to moderate (11 to 14) and severe (15 to 21). 

Using the Mini-Mental State Examination (MMSE),^   10 ^ our psychologist evaluated the patients’ mental process of knowing, including awareness, perception, reasoning, and judgment. The MMSE is a 30-point questionnaire and examines the following functions: registration, attention and calculation, recall, language, ability to follow simple commands, and orientation. The scores of 24-30 are considered normal, and less than 24 as cognitive function impairment. We divided the impaired scores were to mild (17 to 23) and severe (0 to 16).

Statistical data analysis was performed by SPSS software (version 16, SPSS Inc. Chicago, IL, USA). Continuous data were presented as mean ± SD and range, and they were compared using Student t test. Categorical data were shown as counts and percentages, and were compared using chi-square and Fisher’s exact tests. Nonparametric data were compared using nonparametric tests. Significance was considered at a value of less than 0.050.

## Results

Fifty-three patients were entered in the study. Mean age of patients was 50.9 ± 13.6 years (range: 17-73) with 28 women (52.8%) and 25 men (47.2%). Thirty patients (62.3%) were treated by clipping and 20 patients (37.7%) by coiling. Mean follow-up duration of the patients was 17.2 months with the range of 9-48 months. 

**QOL:** Our patients’ mean score of QOL and its different components are shown in table 1. 

**Table 1:** The results of quality of life (QOL) assessment among studied patients with subarachnoid hemorrhage (SAH)

**Variable**	**Total**
QOL	57.8 ± 23.1
Emotional QOL	57.7 ± 23.8
Physical QOL	58.0 ± 25.7
SF	68.1 ± 29.5
MH	58.8 ± 21.4
VT	55.8 ± 24.3
RE	48.1 ± 45.0
GH	60.8 ± 21.8
BP	64.7 ± 28.5
RP	44.8 ± 43.7
PF	61.6 ± 31.0

QOL: Quality of life; SF: Social functioning; MH: Mental health; VT: Vitality; RE: Role limitations due to emotional problems; GH: General health; BP: Body pain; RP: Role limitations due to Physical health; PF: Physical functioning

**Figure 1:**
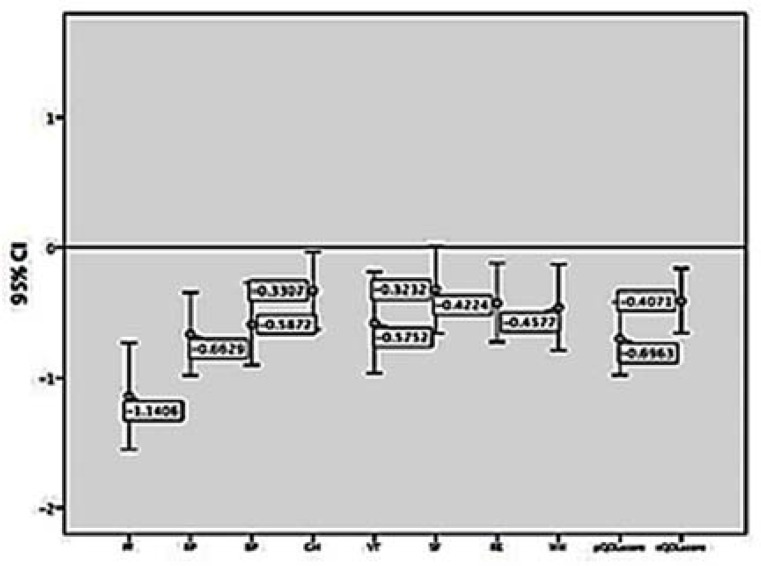
The standard scores of 8 components, physical, and emotional quality of life (QOL)

PF: Physical functioning; RP: Role limitations due to physical health; BP: Body pain; GH: General health; VT: Vitality; SF: Social functioning; RE: Role limitations due to emotional problems; MH: Mental health; CI: Confidence interval

Figure 1 shows that the standard scores are less than the general population in all components. PF achieved the least score, far away from the general population. Our patients were also most affected by role limitations due to physical health, BP, and VT. The physical QOL was affected more than the emotional QOL (Figure 1), but the difference was not statistically significant (P = 0.122). 

***Depression and cognitive function:*** Table 2 shows the status of depression and cognitive function among studied patients. Regarding depression, the mean HADS was 11 in all patients. Twenty-four patients (45.3%) had normal or borderline HADS scores, and it was impaired in 29 patients (55.1%). Patients with depression (impaired HADS) had a worse score of QOL than patients without depression (43.2 vs. 75.5, P < 0.001). 

The median MMSE was 22 in all patients with the range of 10-30. Thirty patients (56.6%) have cognitive impairment, including 12 patients (22.6%) with a severe impairment.

**Table 2:** The status of depression and cognitive function among studied patients with subarachnoid hemorrhage (SAH)

**Questionnaire**		**n (%)**
MMSE	Normal	23 (43.4)
	Mild	18 (34.0)
	Severe	12 (22.6)
HADS	Normal or borderline	24 (45.3)
	Moderate	6 (11.3)
	Severe	23 (43.4)

MMSE: Mini-Mental State Examination; HADS: Hospital Anxiety and Depression Scale

***The effect of gender, age, the location of aneurysm, and the type of treatment:*** Gender of patients did not affect the achieved QOL and its components (P > 0.050 for all). Depression was more common among the women than men, but it did not reach statistical significance (abnormal HADS of 64.3% vs. 44.0%, respectively, P = 0.139). Regarding abnormal MMSE, there was no significant difference between women and men, although more women had impaired MMSE compared to men (67.9% vs. 44.0%, respectively, P = 0.080). 

Despite there was no significant correlation between QOL and age (P > 0.050); older patients affected slightly more in PF (P = 0.061), GH (P = 0.054), and role limitations due to emotional problems (P = 065). The patients with impaired cognitive function were older than those with normal cognitive function (56.6 ± 11.9 vs. 43.4 ± 12.1 years, respectively, P < 0.001). There was no correlation between age and abnormal HADS scores (P = 0.153).

There was no significant difference between the patients with and without anterior communicating aneurysm (AcoA) regarding QOL score and its two components, emotional and physical QOL (P = 0.551, P = 0.910, P = 0.330, respectively). The 8 components of QOL were not significantly different between these two groups except for GH that was better in patients with AcoA (P = 0.041). Although more patients with AcoA suffered from cognitive impairment than those without AcoA, the difference was not significant (60.0% vs. 53.4%, P = 0.305). There was no significant difference for impairment in HADS scores between the patients with and without AcoA (44% versus 54%, P = 0.232). 

Type of treatment (clipping or coiling) did not affect the results of QOL scores and its components (P > 0.050 for all). Patients treated with clipping were not different from patients with coiling regarding depression (P = 0.974) and cognitive dysfunction (P = 0.126).

## Discussion

In this study, we evaluated the QOL, cognitive impairment, and depression among the patients with aSAH 6 to 48 months after their presentation. To assess QOL, patients filled the SF-36 questionnaire by their own, and expressed their own recovery that could be very different from the assessment done by physicians.^        11 ^ SF-36 form include simple questions that can be completed by almost all patients (96%) with aSAH.^        12 ^ The validity and reliability of SF-36 were assessed in the Iranian general population, and the normal values were described before us.^   9 ^ All the components of QOL were less than the general population in our patients, and PF was the most affected component. Other studies also reported an important decrease in all aspects of QOL among the patients with SAH.^5^^,^^7^^,^^12^^-^^14^ Similar to other studies, PF and role limitation for PF were mainly affected in our patients, but in contrast to the other studies,^13^^,^^14^ VT from emotional QOL was mostly affected. In contrast to other studies,^11^^,^^15^ we found that physical QOL was affected more than the emotional QOL. These differences could be expressed by cultural differences between various regions. 

Majority of patients showed a significant improvement in QOL during 10-20 years of follow-up.^16^^,^^17^ Despite the significant improvement of QOL, more than 50% of patients could not integrate completely with society, even after 2 decades.^   17 ^ Several studies found different predictive factors of poor QOL including older age,^12^^,^^18^ female sexuality,^7^^,^^19^ poor neurological status on admission,^12^^,^^19^ severe SAH on admission,^12^^,^^15^^,^^18^^,^^20^ and severe physical disability.^2^^,^^13^^,^^15^^,^^19^^,^^21^^,^^22^ In our study, gender, age, the location of aneurysm, and type of treatment did not affect the result of QOL. But in a meta-analysis study, Noble and Schenk showed that the only important predictive factor of poor QOL was the physical disability.^        11^

In our study, patients with depression had worse QOL than patients without it. This result was consistent with Jonsson, et al. study that reported a great association between QOL and depression in patients with stroke.^23^ We did not find any correlation between the result of QOL and AcoA location except for better GH in patients without AcoA. Haug, et al. reported that patients with AcoA had worse results of several QOL components than patients with middle cerebral artery (MCA) aneurysm including physical role, BP, and GH, and patients with MCA aneurysm had a slightly better functioning.^        24 ^

Similar to other studies,^5^^,^^12^^,^^25^^-^^27^ depression was common in our patients (55%). Depression and anxiety are reported in 17 to 75 percent of patients with aSAH.^27^^,^^28^ There is a slight improvement in mood disorders over time.^        27 ^ Several patients with SAH usually complain of lack of energy and poor motivation that could be related to apathy and depression.^12^ Significant correlation between low serum cortisol and depression and also between low serum cortisol and QOL has been reported by Kreitschmann-Andermahr, et al.^        15 ^ Furthermore, most of the depression and anxiety symptoms could be explained by post-traumatic stress disorders (PTSD).^25^^, , ^^29^ ^30^ About one-third of the patients with SAH are reported to fulfill the criteria of PTSD.^29^^,^^30^ PTSD and depression could explain why QOL of patients with aSAH remains low even after many years, and they cannot return to their work.^29^^,^^31^ Despite the high prevalence of mood disorders in patients with SAH, the high percentage of them do not receive any treatment for their depression, anxiety, and PTSD.^        32 ^ Accordingly, treatment of mood disorders, PTSD, and associated hypopituitarism could improve the functioning of patients with SAH and their integration into normal life and social activity.

Fifty-seven percent of our patients had cognitive impairment and 22.6% were severely affected. We used MMSE for assessment of cognitive impairment, as patients can complete it quickly and easily. MMSE is the most common cognitive screening assessment tool in neurosurgery, and it is a sensitive test for evaluating patients with SAH at discharge and 1 year after discharge.^33^^,^^34^ As 24 to 26 was set for the cut-off point of cognitive impairment in the majority of studies,^33^^,^^34^ we also defined 24 as the abnormal cognition cut-off point. The prevalence of cognitive impairment was reported between 6-59.6 percent.^3^^,^^33^^-^^35^ Verbal memory and motor functioning are the most affected parts, and visual memory, visuospatial function, and exclusive function are the least affected ones.^3^^,^^36^ Cognitive problems can explain the problem of patients with aSAH in returning to work and in integration in society despite their good functional outcomes. Consistent with other studies,^   36 ^ older patients suffered from more cognitive impairment in our study. Other factors like global cerebral edema, left-sided infarction, poor neurological condition on admission, and thick SAH are reported to be associated with cognitive impairment.^   36 ^ As Sonesson, et al. reported,^   17 ^ we did not find any relation between cognitive problem and the location of aneurysm. 

Several studies reported better cognitive function and neuropsychological functioning in patients treated by endovascular methods than patients treated by clipping.^37^^,^^38^ However, we did not find any difference between these two methods of treatment. Frontotemporal lobe damage during surgery could explain the difference between microsurgical and endovascular group.^   37 ^ Despite the concept of better functional and cognitive outcome in endovascular group compare to surgical group, it seems that the impact of SAH on brain and its damage is more important factor for neuropsychological function, cognitive function, and QOL in these patients, and neither the location of aneurysm and nor type of treatment are important in this regard.

***Limitation*** *:* The number of population in our study was limited. The educational status, social activities, and employment status of patients before SAH were not assessed. We used limited neuropsychiatric tests for evaluation of our patients, and more tests with more details could provide better results. The range of follow up in our patients was very wide, that might result in different scores in patients with aSAH who are in the recovery period.

## Conclusion

Our study showed that patients with aSAH suffer from several neuropsychological sequels which could not be assessed by routine functional outcome scales like mRS and GOS. These findings can support the inability of these patients to return to work and integrate into society. Long-term assessment of patients with SAH with appropriate neuropsychiatric tests may help to find their neuropsychological problems, and better management of their problems.

## Notes:

**How to cite this article:** Zabyhian S, Mousavi-Bayegi SJ, Baharvahdat H, Faridhosseini F, Sasannejad P, Salehi M, et al. Cognitive function, depression, and quality of life in patients with ruptured cerebral aneurysms. Iran J Neurol 2018; 17(3): 117-22.
